# Effects of lactoferrin derived peptides on simulants of biological warfare agents

**DOI:** 10.1007/s11274-016-2171-8

**Published:** 2016-11-10

**Authors:** Tjitske Sijbrandij, Antoon J. Ligtenberg, Kamran Nazmi, Enno C. I. Veerman, Jan G. M. Bolscher, Floris J. Bikker

**Affiliations:** Department of Oral Biochemistry, Academic Centre for Dentistry Amsterdam, University of Amsterdam and VU University Amsterdam, 1081 LA Amsterdam, The Netherlands

**Keywords:** Antimicrobial activity, Antimicrobial peptide, Biowarfare simulants, Lactoferrin, LFchimera

## Abstract

Lactoferrin (LF) is an important immune protein in neutrophils and secretory fluids of mammals. Bovine LF (bLF) harbours two antimicrobial stretches, lactoferricin and lactoferampin, situated in close proximity in the N1 domain. To mimic these antimicrobial domain parts a chimeric peptide (LFchimera) has been constructed comprising parts of both stretches (LFcin17–30 and LFampin265–284). To investigate the potency of this construct to combat a set of Gram positive and Gram negative bacteria which are regarded as simulants for biological warfare agents, the effect on bacterial killing, membrane permeability and membrane polarity were determined in comparison to the constituent peptides and the native bLF. Furthermore we aimed to increase the antimicrobial potency of the bLF derived peptides by cationic amino acid substitutions. Overall, the bactericidal activity of the peptides could be related to membrane disturbing effects, i.e. membrane permeabilization and depolarization. Those effects were most prominent for the LFchimera. Arginine residues were found to be crucial for displaying antimicrobial activity, as lysine to arginine substitutions resulted in an increased antimicrobial activity, affecting mostly LFampin265–284 whereas arginine to lysine substitutions resulted in a decreased bactericidal activity, predominantly in case of LFcin17–30.

## Introduction

LF is a multifunctional 80 kDa glycoprotein and secreted as innate immunity factor in secretory fluids, e.g. tears, saliva, and milk and also found in the granules of the neutrophils (Farnaud and Evans 2003). It is known to have bactericidal, fungicidal, and antiviral activity as well as antitumor, anti-inflammatory and immunoregulatory properties (Brock 1995). Most of these activities reside in the basic N1-domain of LF. Lactoferricin-B (LFcinB), released by gastric pepsin cleavage of bovine LF (bLF) (Bellamy et al. 1992), is a positively charged looped peptide consisting of amino acid residues 17–41, with more potent bactericidal and fungicidal activity than the native protein. Studies on a shorter variant, consisting of amino acid residues 17–30 (LFcin17–30), revealed a broad-spectrum bactericidal activity against Gram-positive and Gram-negative bacteria including *Bacillus subtilis, Escherichia coli,* and *Pseudomonas aeruginosa* (Groenink et al. 1999). Besides LFcinB, the N1-domain contains a second stretch, designated LFampin, which also shows features that are characteristic for AMPs, including the presence of positively charged residues and a hydrophobic domain. Previously, two bovine LFampin variants, LFampin268–284 and LFampin265–284, were compared with respect to their bactericidal activities. It was found that LFampin265–284 killed a set of Gram-positive bacteria that were resistant to LFampin268–284. It appeared that the presence of the N-terminal sequence, ^265^Asp-Leu-Ile^268^, enhanced the propensity of LFampin to adopt an α-helix, leading to an improved activity (Bolscher et al. 2006; Van der Kraan et al. 2004).

The mutual orientation of the two antimicrobial stretches localized at the edge of the N1-domain prompted us to investigate the activities in conjunction as well as in separation. Therefore a chimerical structure consisting of LFcin17–30 and LFampin265–284 was generated by linking both peptides to the two amino group of a lysine residue (Bolscher et al. 2009). NMR analysis showed that this lysine linker forced both peptides to extended α-helixes (Haney et al. 2012). This LFchimera exhibited stronger bactericidal activity than its constituent peptides either individually or as a mixture, not hampered by the shielding effects of high ionic strength (Bolscher et al. 2009).

The potency of this chimeric peptide provoked us to test the peptide against various human pathogens, like *Staphylococcus aureus* (Flores-Villasenor et al. 2010) *Escherichia coli* O157:H7 (Flores-Villasenor et al. 2012), *Streptococcus pneumoniae* Leon-Sicairos et al. 2014) and *Burkholderia pseudomallei* (Kanthawong et al. 2009; Puknun et al. 2013, 2016), the latter which is classified by the Centers for Disease Control and Prevention as a category B biological warfare agent (BWA) (Rotz et al. 2002).

However, development of new AMPs for combatting BWA entails several safety, security and logistical drawbacks including culturing and testing of virulent bacteria under adequate bio-containment conditions, hampering large screenings of peptides. In order to overcome these difficulties most of the research activities and tests reported so far, are performed using simulants, BWA simulants (BWA-S), which are phylogenetically or structurally related to BWA. Therefore, for the present study we have chosen a set of both Gram positive and Gram negative bacteria, which are considered BWA-S (Adducci et al. 2016; Bikker et al. 2006; Dawson and Liu 2008; Kaman et al. 2011a). These include *Bacillus cereus* and *Bacillus globigii,* considered safer simulants for *Bacillus anthracis,* the etiologic agent of anthrax (Jansen et al. 2014). Furthermore we included two strains which are closely related to *Yersinia pestis* i.e*. Yersinia enterocolitica* and *Yersinia pseudotuberculosis,* the latter causing severe fever and abdominal pain (Atkinson and Williams 2016; Kaman et al. 2011a). Finally, the effects on two *Salmonella* spp. were studied, including a distinct strain of *Salmonella enterica* serotype *typhimurium*, known as definitive type 104 (DT104), a major cause of gastroenteritis in humans and animals (Hall 2010).

The antimicrobial activities of LFchimera and its constituent peptides as well as the native bLF were mapped by studying (1) the minimum bactericidal concentration (MBC), (2) membrane integrity by using the propidium iodide (PI) assay and (3) membrane polarity using a DiSC3(5) assay. Furthermore we aimed to increase the antimicrobial potency of the bLF derived peptides by cationic amino acid substitutions. As cationic amino acid residues attract the peptide to the anionic bacterial membrane, positively charged residues including arginine (Arg, R), lysine (Lys, K) and histidine (His, H) play an important role within AMPs in general. Despite their identical charge, Arg residues appear more prevalent in naturally occurring AMPs than Lys and His (Cutrona et al. 2015; Henriques et al. 2006; Hristova and Wimley 2011; Joliot and Prochiantz 2004; Yeaman and Yount. 2003). Besides, by in vitro experiments it was found that Lys to Arg substitutions increased the antimicrobial effects of peptides, implying that the guanidinium group in Arg may be preferable for activity than the amine group found in Lys (Cutrona et al. 2015; Strom et al. 2003). In line, we analyzed variants of LFcin17–30, LFampin265–284 and LFchimera in which all Arg residues were replaced for Lys residues and vice versa. Arg to His and Lys to His substitutions were omitted as LF peptides do not contain His.

## Materials and methods

### bLF and LFpeptides

bLF was kindly provided by DMV International (Veghel, The Netherlands). LF derived peptides (Table 1) were synthesized by solid phase peptide synthesis using Fmoc chemistry with a Siro II synthesizer (Biotage, Ippsala, Sweden) according to the manufacturer’s protocol and purification by Reverse Phase-HPLC was conducted as described previously (Bolscher et al. 2009). Identity of the peptides was confirmed by mass spectrometry (Bruker Daltonik GMBH, Bremen, Germany) and molar concentrations were calculated based determined on their weight.

**Table 1:** Sequences and characteristics of the peptides investigated

Peptide^a^	Primary structure	Mol wt	Charge^b^
LFampin265–284	DLIWKLLSKAQEKFGKNKSR	2390	4+
LFampin265–284 all K	DLIWKLLSKAQEKFGKNKSK	2362	4+
LFampin265–284 all R	DLIWRLLSRAQERFGKNRSR	2502	4+
LFcin17–30	FKCRRWQWRMKKLG	1923	6+
LFcin17–30 all K	FKCKKWQWKMKKLG	1839	6+
LFcin17–30 all R	FRCRRWQWRMRRLG	2007	6+
LFchimera		4422	+12
LFchimera all K		4310	+12
LFchimera all R^c^		4618	+12

^a^The purity of the peptides was at least 95% and the authenticity of the peptides was confirmed by ion trap mass spectrometry

^b^Calculated net charge at neutral pH

^c^The lysine linker residue is not replaced by an arginine; this residue does not account to the overall charge of the peptide as the α- and ε-amino-groups are substituted

### Bacteria

The Gram positive *Bacillus cereus* (BM629), *Bacillus globigii* (BM013) and Gram negative *Yersinia enterocolitica* (DSM 4780), *Yersinia pseudotuberculosis* (DSM 8992), *Salmonella typhimurium* SF1399 (BM352) and *Salmonella typhimurium* DT104a (BM638) were cultured overnight aerobically in trypticase soy broth (TSB) medium at 37 °C for *Bacillus* sp. and *Salmonella* sp. or 30 °C for *Yersinia* sp.

### Minimum bactericidal concentration

The killing activities of all peptides and bLF against all bacteria were determined by determining the minimum bactericidal concentration (MBC) as described previously (Kanthawong et al. 2009). Briefly, bacterial cells were washed three times and were re-suspended (approximately 10^5^ CFU/ml) in 1 mM potassium phosphate buffer (PPB), pH 7.0. The bacterial suspension was then added to an equal volume of the tested agents to reach a final concentration ranging from 0.1 to 50 μM. A bacterial suspension in PPB without peptide served as a control. Following incubation at 37 °C for 60 min, the incubation mixture was serially diluted in a physiological concentration of saline and plated in triplicate on TSA. Colonies were counted after 24 h of incubation at 37 °C. A bactericidal effect was defined as a ≥3 log_10_ reduction in CFU/ml compared with the initial inoculum. Each assay was performed on three separate occasions, with duplicate determinations each time.

### Propidium iodide assay

To assess whether the peptides render the bacterial membrane permeable a propidium iodide (PI) assay was performed as described before (Van der Kraan et al. 2004). PI fluorescence is enhanced upon binding to nucleic acids, after passing through a disrupted membrane. Briefly, serial dilutions of peptides or bLF (0.1–12.5 μM) in PPB were incubated with approximately 10^8^ CFUs/ml bacteria suspension and 10 μM PI (Invitrogen, Carlsbad, CA, U.S.A.) for 1 h in a black 96-well plate (Greiner, Recklinghausen, Germany). Increase of fluorescence was monitored using a spectrophotometer (Spectramax M2, Molecular Devices, Sunnyvale, CA, USA) at excitation and emission wavelengths of 535 and 617 nm, respectively. The experiments were performed in duplicate and repeated three times.

### DiSC3(5) assay

Membrane polarity was assessed with 3,3′-Dipropylthiadicarbocyanine iodide (DiSC3(5), ThermoFisher Scientific, Bleiswijk, the Netherlands). The potentiometric probe, DiSC3(5) is a carbocyanine with a short (C3) alkyl tail. This cationic dye accumulates on hyperpolarized membranes and is translocated into the lipid bilayer. DiSC3(5) is quenched at relative high concentrations; fluorescence is increased upon dissipation of the membrane potential (Δψ) by permeabilization.

A bacterial suspension of approximately 10^8^ CFU/ml in PPB was prepared and incubated with 1.5 μM DiSC3(5) and 1 M glucose in a black 96-well plate for 30 min. Next, serial diluted peptide (0–12.5 μM) was added and incubated for 30 min. Increase of fluorescence was monitored using a spectrophotometer (Spectramax M2) at excitation and emission wavelengths of 622 and 655 nm, respectively. The experiments were performed in duplicate and repeated three times.

## Results

### Bactericidal effect of bLF and derived peptides

The bactericidal activity of bLF and the peptides listed in Table 1, were determined on a set of Gram positive and Gram negative bacteria known as simulants for agents potentially involved in biowarfare and bioterrorism (BWA-S) (Table 2). bLF was bactericidal only for the *Bacillus* sp. The LFchimera’s were the most potent peptides showing bactericidal activity against all bacteria and being active in the lowest concentrations compared to the other peptides. The other peptides showed bactericidal activity against *B globigii, B. cereus, Y. pseudotuberculosis,* and both *S. typhimurium* spp, but not against *Y. pseudotuberculosis*.

**Table 2:** Antimicrobial activities of bLF and LF-peptides (MBC, μM) against biological warfare agent simulants

	*B. globigii*	*B. cereus*	*Y. enterocolitica*	*Y. pseudotuberculosis*	*S. typhimurium SF1399*	*S. typhimurium DT104a*
bLF	25	3.1	ND	ND	ND	ND
LFampin265–284	1.6	25	6.3	ND	12.5	6.3
LFampin265–284 all K	1.6	25	6.3	ND	25	12.5
LFampin265–284 all R	0.8	12.5	3.1	ND	3.1	1.6
LFcin17–30	1.6	3.1	0.8	ND	1.6	1.6
LFcin17–30 all K	3.1	25	1.6	ND	1.6	3.2
LFcin17–30 all R	1.6	0.8	0.8	ND	1.6	1.6
LFcin17–30 and LFampin265–284	0.4	3.1	0.8	ND	0.8	0.4
LFchimera	0.4	0.2	0.2	1.6	0.2	0.4
LFchimera all K	0.4	0.2	0.2	6.3	0.2	0.2
LFchimera all R	0.4	0.4	0.2	1.6	0.2	0.4

Bactericidal effect was defined as a ^10^log[reduction] > 3 in viability (Puknun et al. 2013)

*ND* not detected up to 50 μM peptide

Except for *B. globigii* LFcin17–30 showed a stronger bactericidal activity than LFampin265–284. Substitution of Arg residues to Lys residues of both LFcin17–30 and LFampin265–284 resulted in comparable or decreased bactericidal activity. For example, LFampin265–284 all K showed comparable antimicrobial activity against both *Bacilli*, and *Y. enterocolitica*. For the *Salmonellae* spp, the antimicrobial activity of LFampin265–284 all K was slightly decreased compared to LFampin265–284 (Table 2).

Substitution of Lys to Arg residues increased bactericidal activity for LFampin265–284 in all cases. For LFcin17–30, the Lys to Arg substitution increased bactericidal activity against *B. cereus* (3.1 to 0.8 μM), but not against the other bacteria (Table 2).

Except for *B. cereus* and *Y. enterocolitica*, the combination of LFcin17–30 and LFampin265–284 showed a stronger antimicrobial activity than LFcin17–30, the most potent of the two peptides (Table 2). Exclusively the LFchimera’s showed antimicrobial activity against all strains tested, including *Y. pseudotuberculosis*. LFchimera exposed a stronger bactericidal activity compared to the combination of its constituents LFcin17–30 and LFampin265–284, except for *B. globigii,* and *S. typhimurium DT104n* where activity was comparable. For the LFchimera, both Lys to Arg as well as the Arg to Lys substitutions were hardly effective, except for *Y. pseudotuberculosis,* where the Arg to Lys substitution decreased activity; from 1.6 to 6.3 μM (Table 2).

### Effect of bLF and derived peptides on the bacterial membrane permeability

**Fig. 1:**
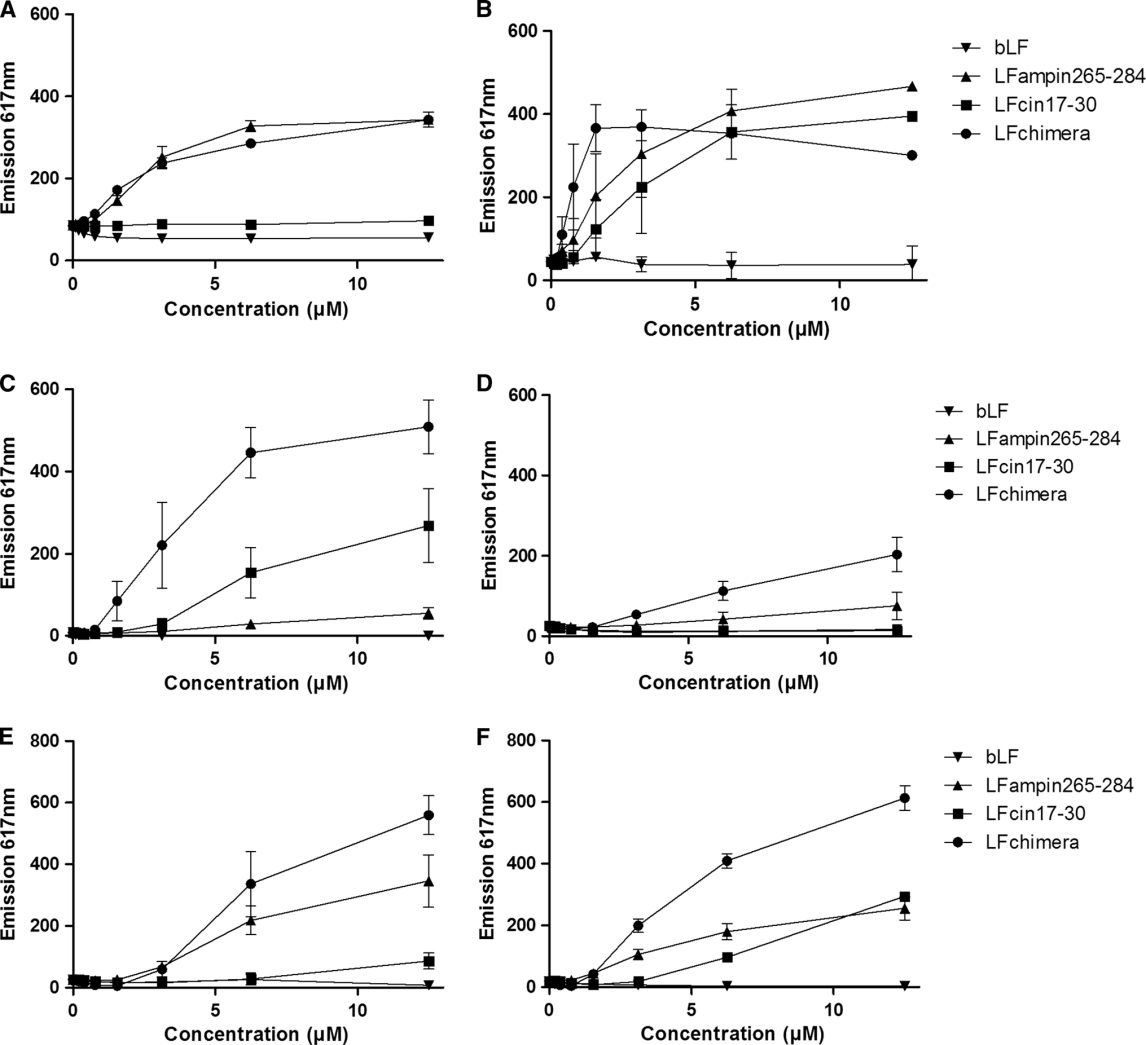
Effect of bLF and bLF-derived peptides on membrane permeabilization, as assessed by the PI assay of *B. globigii* (**a**), *B. cereus* (**b**), *Y. enterocolitica* (**c**), *Y. pseudotuberculosis* (**d**), *S. typhimurium* SF1399 (**e**), and *S. typhimurium* DT104a (**f**). Fluorescence was monitored at an excitation wavelength at 535 nm and an emission wavelength 617 nm. Data are shown as mean ± SEM (*n* = 3)

The effect on the membrane permeability was investigated with the nucleic acid staining fluorescent propidium iodide (PI) (Fig. 1). For all the bacteria tested, the addition of bLF did not lead to an increase in PI fluorescence after 1 h of incubation (Fig. 1). The addition of LFampin265–284 caused relative strong permeabilization of the plasma membrane of *B. globigii, B. cereus,* and both *Salmonella* strains (Fig. 1a, b, e, f). Only relative mild effects were observed for the *Yersinia* sp. (Fig. 1c, d).

LFcin17–30 caused PI fluorescence in *B. cereus* (Fig. 1b), *Y. enterocolitica* (Fig. 1c) and *S. typhimurium* DT104a (Fig. 1f). *B. cereus* seemed the most sensitive bacterium where high PI fluorescence was reached at 6.3 μM. In contrast, *B. globigii* was the least sensitive to LFcin17–30, as no PI fluorescence was detected for concentrations up to 12.5 μM (Fig. 1a).

PI fluorescence in the presence of the LFchimera was comparable to LFampin265–284 for both *Bacillus* spp. (Fig. 1a, b). In case of both *Yersinia* spp. and *Salmonella* spp. the LFchimera caused strongest increase compared to bLF and the other peptides (Fig. 1c, d).

### Effect of peptides on bacterial membrane polarization

**Fig. 2:**
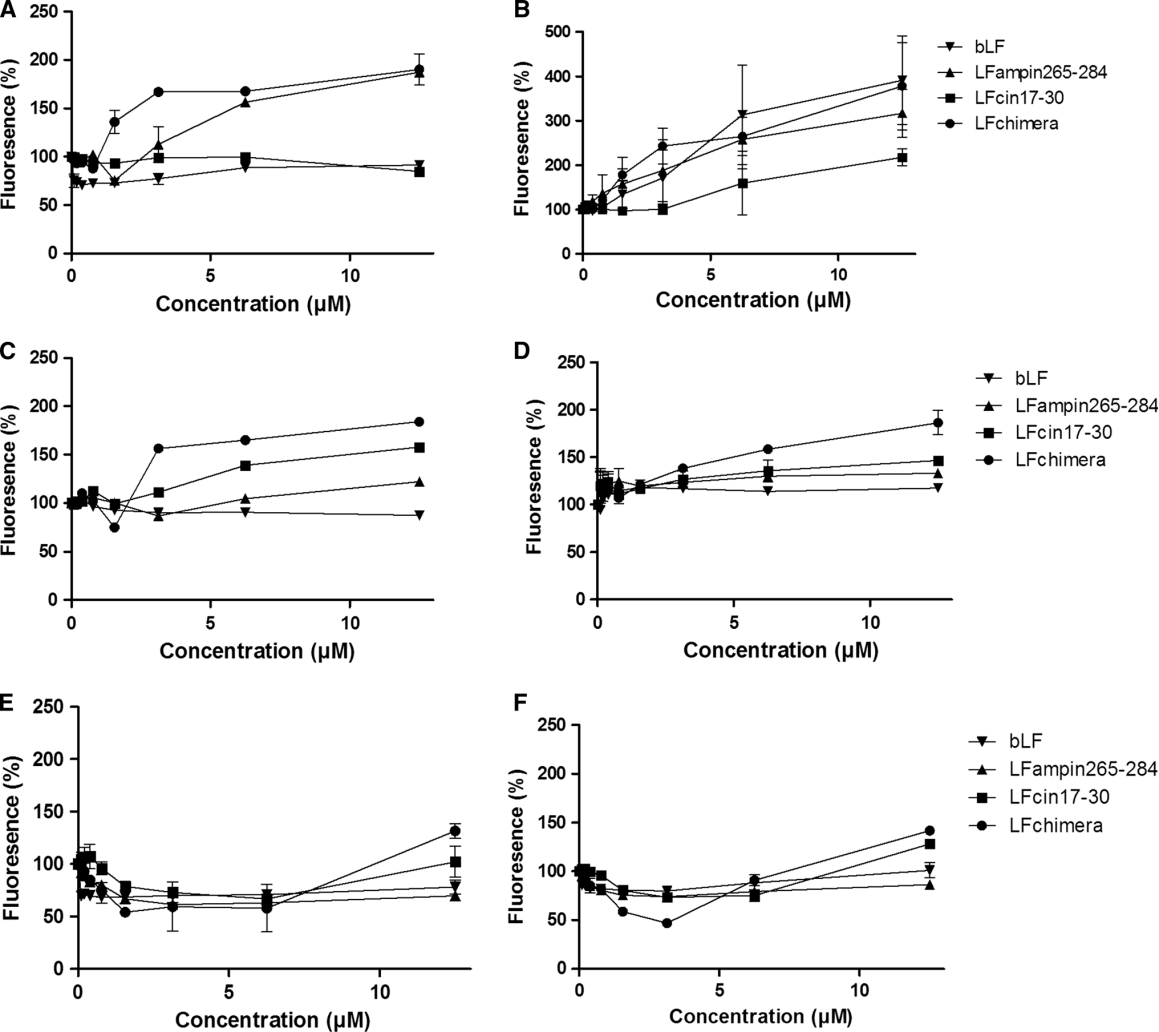
Effect of bLF and bLF-derived peptides on membrane polarity, as assessed by the DiSC3(5) assay of *B. globigii* (**a**), *B. cereus* (**b**), *Y. enterocolitica* (**c**), *Y. pseudotuberculosis* (**d**), *S. typhimurium* SF1399 (**e**), and *S. typhimurium* DT104a (**f**). Data are shown as mean ± SEM (*n* = 3)

To investigate whether membrane damage played a role in the bactericidal activities of bLF and LF derived peptides, we determined membrane depolarization with the membrane potential-sensitive dye DiSC3(5) (Fig. 2). bLF caused an increase in DiSC3(5) fluorescence compared to the control in *B. cereus* (Fig. 2b) but no effects were found with the other bacteria tested. LFampin265–284 caused an increase in DiSC3(5) fluorescence in both *Bacillus* sp. (Fig. 2a, b); at 12.5 μM DiSC3(5) fluorescence increased in *B. globigii* and *B. cereus* to ~200 and ~300%, respectively, compared to the control. For both *Yersinia* sp. increase in DiSC3(5) was relatively mild, i.e. 10–20% (Fig. 2c, d). In both *Salmonella* spp. no increase in DiSC3(5) fluorescence was observed in the presence of LFamp265–284 (Fig. 2e, f). Although LFcin17–30 was in general more bactericidal than LFampin265–284 DiSC3(5) fluorescence was lower. LFcin17–30 increased DiSC3(5) fluorescence up to ~200% for *B. cereus* (Fig. 2b) and 150% in case of *Y. pseudotuberculosis* (Fig. 2d). In all other cases increase in fluorescence was low, i.e. below 10%.

The most profound effect was observed with the LFchimera, especially in case of the *Bacillus* sp. and *Y. enterocolitica*; relative high DiSC3(5) fluorescence was observed in the presence of 3.1 μM peptide. Although less profound in case of *Y. pseudotuberculosis*, the presence of LFchimera was more effective compared to bLF, LFcin17–30 and LFampin265–284, i.e. 180% at 12.5 μM (Fig. 2d). Finally, only at 12.5 μM an increase in fluorescence was observed in the *Salmonella* spp. of approximately 25% (Fig. 2e, f).

## Discussion

One of the aims of the presented study was to map the effects of bLF derived peptides on bacterial killing, membrane polarity and membrane permeability on a set of Gram positive and Gram negative bacteria which can be regarded as ‘safe-to-handle’ simulants for BWA. In addition we studied the effects of cationic amino acid substitutions of the LF peptides in order to enhance the antimicrobial potency and to better understand underlying mechanisms.

In general, iron sequestration, leading to inhibition of microbial growth, is regarded as the main antimicrobial effect of LF (Brock. 1995). In the present study bLF showed direct bacterial killing activity on both *Bacillus* spp. with *B. cereus* being the most sensitive (Table 2). Despite the fact that no increase in PI fluorescence was detected for both *Bacillus* spp. (Fig. 1a, b), strong DiSC3(5) release was measured with *B. cereus,* detectable from 1.6 μM bLF (Fig. 2b). This suggests that a disturbance of the membrane potential, and not the membrane leakage, is part of the underlying mechanisms.

The addition of LFampin265–284 and LFchimera caused relativly strong permeabilization (Fig. 1a, b) and depolarization (Fig. 2a, b) of the plasma membrane of *B. globigii* and *B. cereus*. Also in case of LFcin17–30 moderate effects of membrane permeabilization and depolarization ran in parallel showing that membrane permeabilization and depolarization are both related to disturbance of membrane integrity (Figs. 1a, 2a). No membrane effects of LFcin17–30 were observed for *B. globigii* (Figs. 1b, 2b). Yet the MBC for LFampin265–284 and LFcin17–30 was comparable, i.e. 1.6 μM (Table 2). This apparent discrepancy is not fully understood, but it can be hypothesized that differences in LFcin17–30 susceptibility can be explained by other factors e.g. differences in growth speed, membrane fluidity, membrane lipid composition or composition of secreted components such as virulence factors or proteases specificity that favor LFcin17–30 activity against *B. cereus* over *B. globigii* (Kaman et al. 2011b; Teixeira et al. 2012; Ultee et al. 1998). Possibly, LFcin17–30 killed *B. globigii* by a different mechanism, possibly inhibition of RNA synthesis, as was found in *B. subtilis* at sublethal concentrations (Ulvatne et al. 2004).

In general, disturbance of membrane integrity by the presence of LFampin265–284, LFcin17–30 and the LFchimera was in line with the MBC for the different species displaying, low, moderate and high activity, respectively (Table 2) supporting the concept that membrane permeabilization and depolarization are coupled processes leading to bactericidal effects in vitro. An exception was *S. typhimurium* SF1399, which showed more membrane permeabilization appeared higher for LFampin265–284 than for LFcin17–30.

For all bacteria the LFchimera was the most potent bactericidal peptide. Also the membrane disturbing effects of LFchimera were more profound than those of LFampin265–284 and LFcin17–30 (Figs. 1, 2). This effect was most visible for *Y. pseudotuberculosis* where LFamin265–284 and LFcin17–30 were not bactericidal at all (Table 2) and had almost no effect on its membrane integrity (Figd. 1d, 2d). These data strongly support earlier findings; for a broad spectrum of bacteria the LFchimera exhibits stronger bactericidal activity than its constituent peptides either individually or as a mixture (Bolscher et al. 2009, 2012). For example, recently the antimicrobial activities of the LFchimera were tested against isolates of *Burkholderia pseudomallei* and compared to the preferential antibiotic of use ceftazidime (CAZ). All isolates including *B. pseudomallei* 979b shown to be resistant to CAZ, could be killed by 5–10 μM of LFchimera within 2 h, while CAZ, and LFcin17–30 and LFampin265–284 individually only inhibited the *B. pseudomallei* strains, still resulting in an overgrowth in 24 h (Puknun et al. 2016).

Furthermore we aimed to increase the antimicrobial potency of the bLF derived peptides by cationic amino acid substitutions (Nakase et al. 2012). For this we were inspired by work of Cutrona et al. (2015) who compared the activity of histone-derived AMPs: buforin II, DesHDAP1, and parasin with variants that contain only Lys or Arg cationic residues. For all peptides tested, antibacterial activity improved with an increased Arg content; it increased permeabilization for parasin while it improved translocation for buforin II and DesHDAP1 over experimental vesicles. In line, Strom and co-workers found that highest antibacterial activities towards *Escherichia coli a*nd *Staphylococcus aureus* were found using small peptides contained with Arg residues. Vice versa it was found that the replacement of Arg by Lys led to less-active peptides (Strom et al. 2003). However, this effect does not seem ubiquitous as other reports reveal that the introduction of Arg residues do not improve their antimicrobial activity; amino acid replacements may toggle other physical properties e.g. leading to reduction of amphipathicity or alterations of the tertiary structure by a reduced number of intermolecular interactions (Haney et al. 2012; Chen et al. 2003; Nguyen et al. 2011).

We analyzed variants of LFcin17–30, LFampin265–284 and LFchimera in which all Arg residues were replaced for Lys residues and vice versa (Table 1). In line with our expectations Arg to Lys substitutions resulted in an increase in antimicrobial activity compared to the parent peptides (Table 2). Lys to Arg substitutions of LFampin265–284 was most affected. For all five bacteria that were sensitive for LFampim265–284, the variant LFampin265–284 all R with four substituted amino acids, showed increased antimicrobial activity. For LFcin17–30 all R with three substituted amino acids compared to the parent peptide, increased activity was found against *B. cereus.* Reduced activity by Lys to Arg substitutions was not observed. In contrast, Arg to Lys substitutions resulted in a decreased activity compared to the parent peptides. Especially in case of LFcin17–30 all K were four Arg residues were replaced decreased activity was found for *B. globigii, B. cereus, Y. enterocolitica* and *S. typhimurium DT104a.* Similarly, Arg residues were also found to be crucial LFcin17–30 in displaying antimicrobial activity (Silva et al. 2012).

In summary Lys to Arg substitutions resulted in an increased antimicrobial activity, affecting mostly LFampin 265–284 whereas Arg to Lys substitutions resulted in a decreased activity, particularly in case of LFcin 17–30.
